# The intrathecal, polyspecific antiviral immune response in neurosarcoidosis, acute disseminated encephalomyelitis and autoimmune encephalitis compared to multiple sclerosis in a tertiary hospital cohort

**DOI:** 10.1186/s12987-015-0024-8

**Published:** 2015-12-13

**Authors:** Tilman Hottenrott, Rick Dersch, Benjamin Berger, Sebastian Rauer, Matthias Eckenweiler, Daniela Huzly, Oliver Stich

**Affiliations:** Department of Neurology and Neurophysiology, University Medical Center Freiburg, Breisacher Strasse 64, 79106 Freiburg, Germany; ravo Diagnostika GmbH, Oltmannsstrasse 5, 79100 Freiburg, Germany; Department of Neuropediatrics and Muscle Disorders, University Medical Center Freiburg, Mathildenstrasse 1, 79106 Freiburg, Germany; Institute of Virology, University Medical Center Freiburg, Hermann-Herder-Strasse 11, 79104 Freiburg, Germany

**Keywords:** Multiple sclerosis, Intrathecal polyspecific antiviral immune response, MRZ reaction, Neurosarcoidosis, Acute disseminated encephalomyelitis, Autoimmune encephalitis

## Abstract

**Background:**

A polyspecific, intrathecal humoral immune response against the neurotropic viruses, measles, rubella and varicella zoster virus, called “MRZ reaction” (MRZR), is present in the majority of patients with multiple sclerosis (MS). Neurosarcoidosis (NS) and acute disseminated encephalomyelitis (ADEM) are important clinical differential diagnoses of MS. Autoimmune encephalitis (AIE) represents a well characterized autoimmune CNS disorder with intrathecal antibody synthesis. The aim of this study was to investigate the specificity of MRZR for MS in patients with NS, ADEM and AIE for the first time, and to compare it with the diagnostic value of oligoclonal bands (OCB).

**Patients and methods:**

Twenty-two patients with NS, 17 with AIE, 8 with ADEM and 33 with MS serving as controls were analyzed for OCB and MRZR by calculation of the antibody index (AI) for each virus. MRZR was considered as positive if at least two AIs were ≥1.5.

**Results:**

A positive MRZR was statistically significantly less frequent in NS (9 %), AIE (11 %) and ADEM (0 %) compared to MS patients (70 %; *p* < 0.001 each). The specificity of MRZR for MS was 92 % in the study cohort. In comparison to MRZR, the OCB showed a higher sensitivity (100 %), but a lower specificity (69 %) for MS.

**Conclusion:**

These results indicate that MRZR seems to be the most specific available CSF marker of MS.

## Background

In 1992, Felgenhauer et al. first described the polyspecific, intrathecal humoral immune response against the most frequent three antigens, measles (M), rubella (R) and varicella zoster virus (Z), the ‘MRZ reaction’ (MRZR), as being highly specific for multiple sclerosis (MS) [1]. A positive MRZR is characterized by positive antibody indices (AI ≥ 1.5) for these three viruses, indicating a polyspecific, intrathecal humoral immune response [1]. If, unlike in this study, MRZR is defined as at least one positive AI, more than 80 % of MS patients displayed a positive MRZR [1, 2]. The pathophysiological role of the MRZR in MS still remains elusive. Most likely it represents a polyspecific B cell activation (so-called ‘bystander’ reaction) within the CNS, because simultaneous infection with several neurotropic viruses is very unlikely, and polymerase chain reaction (PCR) tests failed to demonstrate viral DNA in CSF from MRZR-positive MS patients [3]. Oligoclonal bands (OCB) represent a sensitive CSF marker of MS, occurring in 90–98 % of patients throughout the course of the disease [4]. However, OCB are also positive in infectious CNS disorders, such as neuroborreliosis (OCB in 70 % [5]), and in autoimmune CNS disorders such as neuromyelitis optica (NMO; OCB in >30 % [6]), and thus are much less specific than MRZR.

Apart from MS, prevalence of MRZR has been studied in two other autoimmune CNS diseases: paraneoplastic neurological disorders (PND) [7] and NMO [8]. Additionally, there is only one case series reporting positive MRZR in a very few patients with systemic autoimmune disorders with CNS involvement including one patient with neurosarcoidosis (NS), three patients with systemic lupus erythematosus and one patient each with Wegener`s granulomatosis and Sjögren syndrome [9].

Occasionally, especially using the revised McDonald criteria [10], the correct diagnosis of MS at initial clinical presentation can be difficult, particularly if there are hints towards differential diagnoses such as NS or acute disseminated encephalomyelitis (ADEM). In such clinical situations, it would be helpful to know how well the MRZR distinguishes between MS and these differential diagnoses. From a more pathophysiological point of view, Jarius et al. questioned in 2009 if the MRZR is specific to MS at all or should rather be considered as a general marker for CNS autoimmune diseases [11]. In this regard, autoimmune encephalitis (AIE) is a well characterized example of CNS autoimmunity suitable for further investigation of the specificity of MRZR. The present study is believed to be the first to systematically address the frequency of a positive MRZR in NS, ADEM and AIE.

## Methods

### Patients

For the purpose of this retrospective study, NS, AIE and ADEM patients treated either at the Department of Neurology or at the Department of Neuropediatrics and Muscle Disorders, University Medical Center Freiburg in Germany, from 2005–2014, were identified through a systematic search of clinic databases. An MS control group was derived from the control group (18 OCB positive MS patients) from a previous study [11], augmented to sufficient size (n = 33) by random selection from all MS patients treated at the same clinic over the same period. Patients were only enrolled if CSF/serum samples were still available after completion of all clinically necessary tests. Diagnoses of MS, NS and ADEM were established according to internationally accepted consensus criteria (MS [10]; NS [12]; and ADEM [13]) and after careful exclusion of relevant differential diagnoses. AIE was diagnosed in the presence of subacute clinical features typical for limbic encephalitis (seizures, affective or memory disorders) and detection of a well characterized IgG antibody against neuronal surface proteins (such as voltage-gated potassium channel (VGKC), *N*-methyl-d-aspartate receptor (NMDAR), gamma-aminobutyric acid B receptor (GABA_B_-R) or glutamic acid decarboxylase (GAD)) and after careful exclusion of relevant differential diagnoses. Lumbar puncture (LP) was performed with the written consent of all patients. As LP was performed for the purpose of initial diagnosis, almost all patients were untreated at the time of LP. CSF and serum samples were taken on the same day and stored according to consensus protocol for the standardization of cerebrospinal fluid collection and biobanking [14]. Haemolytic CSF specimens were excluded. Data concerning patients’ immunization status were not available. All NS, AIE and ADEM patients were included who matched these criteria. The ethics committee of the University Medical Center Freiburg approved the study.

### Determination of MRZR and OCB

IgG concentrations in the serum and CSF were determined nephelometrically (ProSpect System, Siemens, Germany) and Measles-, Rubella- and Varicella-IgG levels in the CSF and serum were measured by enzyme-linked immunosorbent assay (Serion classic ELISA, Germany), both according to the manufacturer’s instructions. Assessment of the MRZR was performed with analysis of virus-specific AIs against M, R, and Z in the Department of Virology, University of Freiburg, Germany according to Reiber’s formula [15]. An AI is a mathematical parameter to assess whether antibodies detected in CSF are derived from blood and have diffused through the blood-CSF barrier (low AI; e.g. <1.5) or have been intrathecally produced. In this study, a virus-specific AI ≥1.5 was considered as indicative of intrathecal antibody production against the respective virus, M, R, or Z. The MRZR was assessed as positive if at least two AIs indicated intrathecal virus-specific antibody production, a definition which has been used by a number of researchers [8, 9, 16, 17]. If an AI could not be calculated due to non-detectable antibodies in the CSF, AI was graded as one.

Detection of OCB was performed in a specialized routine laboratory (Department of Neurology, Freiburg), using a high-sensitive isoelectric focusing technique on agarose gel followed by immunofixation (Hydragel Isofocusing, Sebia, France) as described earlier [11]. Absence of OCB was assumed if there was ≤1 OCB exclusive in CSF.

Statistical comparisons of MRZR results between groups were performed using Fisher’s exact test (two-tailed). Mean AIs of M, R and Z were compared between study groups using the Kruskal–Wallis test with Dunn’s test. A *p* value <0.05 was regarded as statistically significant.

## Results

Of the total population in the database of first-diagnosis NS (n = 201), AIE (n = 25) and ADEM (n = 41), many patients were excluded due to unsure diagnosis or where the diagnosis was later corrected (NS: n = 169, AIE: n = 0 and ADEM: n = 28). Of the remaining patients, there was not enough CSF/serum available for determination of MRZR in a few patients (NS: n = 10, AIE: n = 6 and ADEM: n = 5). Finally, 22 patients with NS, 19 with AIE and 8 with ADEM were analyzed for MRZR. Thirty-three patients with MS served as a control group. Demographic and clinical data of all study patients are presented in Table 1.

**Table 1:** Demographic and clinical data of enrolled patients

Diagnosis	Multiple sclerosis	Neurosarcoidosis	Autoimmune encephalitis	Acute disseminated encephalomyelitis
n	33	22	19	8
Mean age in years (range; SD)	47.8 (23–73; 11.8)	54.7 (31–83; 15.0)	56.2 (31–84; 16.7)	30.6 (4–63; 17.8)
Gender, females in %	69.7	40.9	36.8	50.0
Additional clinical details	MS types: RRMS: n = 14 SPMS: n = 5 PPMS: n = 14	Most frequent neurologic/radiologic syndromes: myelitis: n = 6 subcortical cerebral lesions: n = 6 hydrocephalus: n = 3 brainstem nerve palsy: n = 3 meningitis: n = 2	Detected antibodies against: VGKC: n = 12 NMDAR: n = 5 GAD: n = 2 GABA_B_-R :n = 1 (1 patient had VGKC- and NMDAR-antibodies)	Neurologic syndromes in addition to encephalopathy: brainstem syndrome: n = 3 hemisyndrome due to cerebral lesions: n = 3 cerebellar syndrome: n = 1 myelitis: n = 1

*n* number of patients, *SD* standard deviation, *RRMS* relapsing-remitting multiple sclerosis, *SPMS* secondary progressive multiple sclerosis, *PPMS* primary progressive multiple sclerosis, *VGKC* voltage-gated potassium channel, *NMDAR* *N*-methyl-d-aspartate receptor, *GAD* glutamic acid decarboxylase *GABA*_*B*_-*R* γ-aminobutyric acid B receptor

There were some demographic differences between the four groups, e.g., more women within the MS group and younger ADEM patients. Due to non-detectable antibodies in the CSF, some AIs were graded as one (applicable to 9/99 AIs of MS patients, 5/66 AIs of NS patients, 11/57 AIs of AIE patients and 8/24 AIs of ADEM patients).

**Fig. 1:**
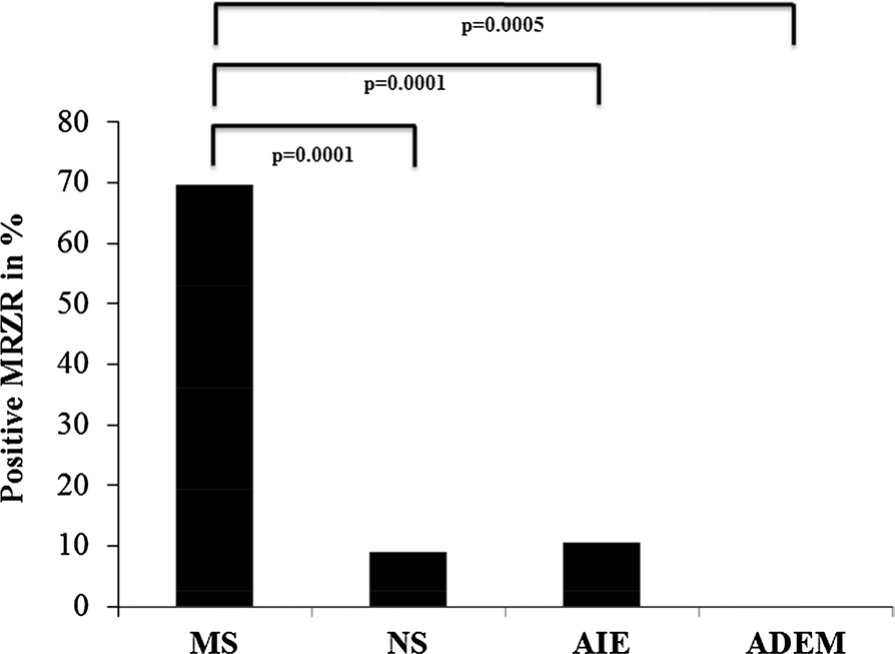
Frequency (in %) of positive measles, rubella and varicella zoster virus MRZR in patients with multiple sclerosis (*MS*: n = 33), neurosarcoidosis (*NS*: n = 22), autoimmune encephalitis (*AIE*: n = 19) and acute disseminated encephalomyelitis (*ADEM*: n = 8). Fisher’s exact test (two-sided)

**Fig. 2:**
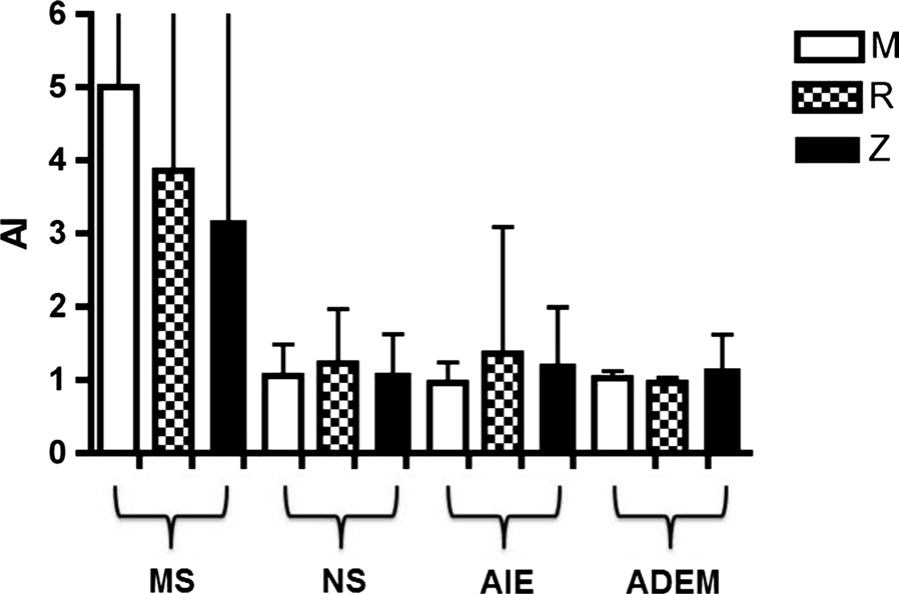
Antibody indices (AIs) for measles (*M*), rubella (*R*), and varicella zoster (*Z*) in patients with multiple sclerosis (*MS*: n = 33), neurosarcoidosis (*NS*: n = 22), autoimmune encephalitis (*AIE*: n = 19) and acute disseminated encephalomyelitis (*ADEM*: n = 8). Standard deviation (SD) of *MS*: M = 7.9, R = 4.9, Z = 3.4

The majority of MS patients (70 %) showed a positive MRZR (16/33 had two positive AIs and 7/33 all three). In contrast, a positive MRZR was much less frequent in patients with NS (9 %; *p* = 0.0001; 1/22 with two positive AIs and 1/22 all three), AIE (11 %; *p* = 0.0001; 2/19 with two positive AIs) and ADEM (0 %; *p* = 0.0005) as presented in Fig. 1. Accordingly, specificity of MRZR for MS was 91.5 % and likelihood ratios were 8.2 (LR+) and 0.3 (LR−). Mean AI values for M, R and Z in NS, AIE and ADEM were all less than 1.5 (range 0.4–8.4, SD 0.8) whereas the MS group revealed mean AI values greater than 3.0 for all three viruses (range 0.5–40.0, SD 5.6) as shown in Fig. 2. Among the 49 non-MS patients, only 3 AIs (representing 2 % of the entire 147 non-MS MRZ-AIs) exceeded 3, and 13 AIs (9 %) lay between 1.5 and 3.0. AIs for R of NS/AIE/ADEM patients, AIs for M of AIE/ADEM patients and AIs for Z of NS patients were statistically significantly lower compared to MS patients. No other statistically significant differences between AIs of MS patients and non-MS patients were found.

All MS patients and 31 % of non-MS patients showed OCB in CSF (OCB prevalence in NS 41 %, AIE 32 % and ADEM 0 %), which corresponds to a specificity of OCB for MS of 69 % in this study cohort.

## Discussion

To our knowledge, this is the first systematic study describing a high MRZR specificity for MS (92 %) in patients with NS, ADEM and AIE. The MRZR sensitivity found here, 70 %, is in line with the two largest previous studies (72 % according to Felgenhauer [1] and 67 % according to Reiber [2]) if the same MRZR definition (at least two positive AIs) is applied to their data. In this study, AIs for MRZ in NS, AIE and ADEM were consistently lower than the values of MS patients, although in the small sample not all differences reached statistical significance. Should a single MRZ-AI be considered, according to our results, an AI value between 1.5 and 3 is not highly specific for MS; whereas an AI >3.0 would reliably support the diagnosis of MS in this clinical context (CNS infection with the respective virus is very unlikely or excluded). Apart from that, MS patients usually show more than one positive MRZ-AI.

As expected, OCB were more frequent in MS patients, but less specific compared to MRZR. Considering the very low rate of a positive MRZR in infectious CNS diseases, such as neuroborreliosis [18] or viral myelitis [19], and other autoimmune CNS disorders, such as NMO [9] or PND [8], these results provide evidence of particularly high specificity of MRZR compared to other diagnostic tools. Reasons for the high MS specificity of MRZR are enigmatic and remain to be addressed in pathophysiological studies.

MRZR is not yet required in the standard diagnostic procedure for patients suspected of MS. This is most likely due to costs and reduced significance of CSF analysis in the 2010 revised McDonald criteria, which has been critically discussed by a group of European authors [20]. The findings of the present and earlier studies lend weight to the proposal that MRZR become part of the recommended diagnostic procedure in cases of suspected MS. Difficulties in ruling out MS mimics (e.g., ADEM, NS and NMO) are not uncommon, as these disorders can reveal similar MRI and routine CSF findings (including OCB). Because these MS mimics require substantially different treatment and have different prognoses, it is crucial not to misdiagnose them as MS [21–23]. Although ADEM often is considered in pediatric patients, MRZR can be used for distinction from MS since it was found to be already positive in the majority of prepubertal MS patients [24]. Determination of MRZR might be helpful in this context due to its high specificity as shown in this and previous studies [7, 8].

The clinic database includes neurosarcoidosis in the more general diagnostic category “sarcoidosis affecting other localizations than lungs, lymph nodes or skin”. Therefore, many patients in this category had to be excluded who showed no involvement of the nervous system. None of the AIE patients had to be excluded due to incorrect diagnosis, most likely as a consequence of searching only for patients with a proven well defined antibody, which obviously enables very high diagnostic certainty.

There are some limitations of this study. First, age and gender of the four groups (MS, NS, AIE and ADEM) were not well balanced due to enrolling unmatched patients because of the rarity of the last three disorders. Second, a selection bias in the monocentric tertiary hospital cohort is conceivable. Third, in view of the small sample size, the retrospective design and the lack of immunization status information, the results should be tested further. A larger cohort of ADEM patients would be advantageous, as would tests in other areas of the world. Extending studies geographically is important because the prevalence of positive AIs differs with immunization status, as evidenced by the lower frequency of intrathecal rubella antibody synthesis in Cuban MS patients [25].

## Conclusions

This study found MRZR to have a specificity of more than 90 % for MS, underlining its high potential as a relevant diagnostic marker in clinical practice. Future systematic investigations of MRZR in patients with other challenging differential diagnoses of MS, such as CNS vasculitis or CNS lymphoma, might be helpful, although for these rare diseases biopsy will remain the diagnostic reference standard.
